# Complete Genome Sequence of *Serratia marcescens* SmUNAM836, a Nonpigmented Multidrug-Resistant Strain Isolated from a Mexican Patient with Obstructive Pulmonary Disease

**DOI:** 10.1128/genomeA.01417-15

**Published:** 2016-01-21

**Authors:** Luisa Sandner-Miranda, Pablo Vinuesa, Gloria Soberón-Chávez, Rosario Morales-Espinosa

**Affiliations:** a Facultad de Medicina, Universidad Nacional Autónoma de México, Mexico City, Mexico; b Centro de Ciencias Genómicas, Universidad Nacional Autónoma de México, Mexico City, Mexico; c Instituto de Investigaciones Biomédicas, Universidad Nacional Autónoma de México, Mexico City, Mexico

## Abstract

*Serratia marcescens* SmUNAM836 is a multidrug-resistant clinical strain isolated in Mexico City from a patient with chronic obstructive pulmonary disease. Its complete genome sequence was determined using PacBio RS II SMRT technology, consisting of a 5.2-Mb chromosome and a 26.3-kb plasmid, encoding multiple resistance determinants and virulence factors.

## GENOME ANNOUNCEMENT

*Serratia marcescens* is emerging as an opportunistic nosocomial pathogen causing urinary tract, bloodstream, skin, and respiratory infections (1). Several virulence factors have been identified, such as hemolysins, proteases, and siderophores (2). Many clinical isolates of this species display intrinsic or acquired resistance to multiple antibiotic families (1).

Here we present the complete genome sequence of *Serratia marcescens* strain SmUNAM836, a nonpigmented, multidrug-resistant bacterium isolated from the bronchial aspiration of a hospitalized patient with chronic obstructive pulmonary disease in Mexico City, as detailed in BioProject accession no. PRJNA284857. Strain SmUNAM836 was resistant to diverse β-lactams, including penicillins, but it is susceptible to cephalosporins and carbapenems. It is resistant to aminoglycosides (tobramycin), fluoroquinolones (ciprofloxacin), and polymixin B.

The genomic DNA of the strain was purified with the DNeasy blood and tissue kit (Qiagen) and sent to the Yale Center for Genome Analysis (YCGA) for PacBio RS II single-molecule real-time (SMRT) sequencing. A standard library of 10-kb fragments was prepared and sequenced on three SMRT cells with the P2-C4 chemistry. The continuous long reads were assembled using the HGAP/Quiver protocol in SMRT portal v.2.3.0.140936.p4 (3), resulting in an assembly with 2 contigs. They were circularized by trimming the terminal repeats with Minimus2 (4) and subjected to two consecutive rounds of read remapping with the RS_Resequencing.1 module for sequence polishing. The final assembly had a mean coverage of ~172× and consists of a chromosome (5,207,023 bp), and a plasmid (26,346 bp), with a mean G+C content of 59.71%. Gene calling and annotation were performed with a modified version of Prokka (5). A total of 4,961 genes were annotated: 4788 CDSs, 95 tRNAs, 22 rRNAs, and 1 tmRNA. Additionally, 45 ncRNAs, 3 riboswitches, and 502 signal peptides were also identified, but no clustered regularly interspaced short palindromic repeat (CRISPR) arrays. The annotation was manually curated and enriched by adding 6 prophage predictions made by the PHAST server (6) and 13 genomic islands identified by IslandViewer3 (7).

The genome was screened for the presence of antibiotic resistance genes, efflux pumps, and potential virulence factors using blastp (8) searches against locally maintained versions of the CARD (9), ResFinder (10), and VirulenceFinder (11) databases. Eight resistance-nodulation-division (RND), two ATP-binding cassette (ABC), and one major-facilitator-superfamily (MFS) type efflux pumps were identified, along with the chromosomally encoded BlaKPC-2 (class B) and AmpC-like (class C) beta-lactamases, the aminoglycoside acetyltransferase Aac(6′)-Ic, and the aminoglycoside phosphotransferase StrA, an QnrB31-like fluoroquinolone resistance protein. No class 1 or class 2 integrons or IS*CR* elements were found.

The plasmid had a low G+C content (43.52%) and displayed ~97% sequence identity with diverse *Enterobacter cloacae* plasmids like pKPC-f91. It harbors the transposon Tn*3* encoding 3 proteins: a BlaTEM-1D (class A) beta-lactamase and the Tn*3* transposase and resolvase. It encodes the genes for the *ccdAB* toxin-antitoxin system.

Potential virulence factors detected are long polar fimbriae (LpfA), hemolysin D (HlyD), and the ClpP protease, the IroN enterobactin siderophore receptor, hemolytic phospholipase C (PlcH), and plasmid-encoded catalase peroxidase (KatP) and enterotoxin (SenB).

### Nucleotide sequence accession numbers. 

The complete genome sequence of *Serratia marcescens* SmUNAM836 is available in GenBank under accession numbers CP012685 (chromosome) and CP012686 (plasmid), BioProject accession number PRJNA284857, and BioSample accession number SAMN03733572.
